# Effects of Hydrogen Peroxide on Wound Healing in Mice in Relation to Oxidative Damage

**DOI:** 10.1371/journal.pone.0049215

**Published:** 2012-11-13

**Authors:** Alvin Eng Kiat Loo, Yee Ting Wong, Rongjian Ho, Martin Wasser, Tiehua Du, Wee Thong Ng, Barry Halliwell

**Affiliations:** 1 Department of Biochemistry, National University of Singapore, Singapore, Singapore; 2 Graduate School for Integrative Sciences & Engineering, National University of Singapore, Singapore, Singapore; 3 Imaging Informatics Division, Live-Cell Imaging and Automation of Image Analysis Group Bioinformatics Institute (BII), Agency for Science, Technology and Research (A*STAR), Singapore, Singapore; 4 Department of Biological Sciences, National University of Singapore, Singapore, Singapore; 5 School of Engineering, Nanyang Polytechnic, Singapore, Singapore; University of Valencia, Spain

## Abstract

It has been established that low concentrations of hydrogen peroxide (H_2_O_2_) are produced in wounds and is required for optimal healing. Yet at the same time, there is evidence that excessive oxidative damage is correlated with poor-healing wounds. In this paper, we seek to determine whether topical application of H_2_O_2_ can modulate wound healing and if its effects are related to oxidative damage. Using a C57BL/6 mice excision wound model, H_2_O_2_ was found to enhance angiogenesis and wound closure at 10 mM but retarded wound closure at 166 mM. The delay in closure was also associated with decreased connective tissue formation, increased MMP-8 and persistent neutrophil infiltration. Wounding was found to increase oxidative lipid damage, as measured by F_2_-isoprostanes, and nitrative protein damage, as measured by 3-nitrotyrosine. However H_2_O_2_ treatment did not significantly increase oxidative and nitrative damage even at concentrations that delay wound healing. Hence the detrimental effects of H_2_O_2_ may not involve oxidative damage to the target molecules studied.

## Introduction

Various groups have shown that H_2_O_2_ plays an important role in wound healing. Non-phagocytes have been shown to produce H_2_O_2_ after wounding which can attract neutrophils [1] as well as promote reinnervation of the peripheral sensory axons [2] in a zebrafish model of wound healing. H_2_O_2_ and O_2_^.-^ have also been detected in mouse wounds [3], [4]. Removal of H_2_O_2_ by catalase over-expression in mice has been reported to delay wound closure and retard angiogenesis [3].

Unsurprisingly, there have been suggestions that application of low levels of H_2_O_2_ may be beneficial for wound healing [5]. Collagen film dressings that contained glucose oxidase were found to promote wound healing in a rat diabetic model apparently by increasing levels of reactive oxygen species (ROS) in the wounds [6]. Glucose oxidase is an enzyme that oxidizes glucose to gluconic acid with the formation of H_2_O_2_ as a by-product. Medicinal grade honey, which has been claimed to promote healing of chronic wounds [7] has also been shown to contain H_2_O_2_, possibly again by the action of glucose oxidase [8].

On the other hand, excessive ROS have been thought to be involved in the pathogenesis of chronic wounds [9]. ROS can cause damage by reacting with nucleic acids, protein and lipids, inducing a loss of function and tissue damage. As ROS, including H_2_O_2_, are inherently damaging, perhaps low concentrations of H_2_O_2_ would promote healing by acting as a signaling molecule while high concentrations would delay healing by causing oxidative damage. Although this hypothesis sounds attractive and simple, it has never been rigorously tested. In fact, the effects of oxidative damage on wound healing have not been fully investigated.

Although it is known that ROS are produced after wounding, little is known about the changes in oxidative damage during wound healing. From clinical studies, chronic wound fluids have been shown to have higher levels of F_2_-isoprostanes, an established marker of lipid peroxidation, than acute wound fluids [10]. Protein oxidation, as measured by protein carbonyls, has also been measured in wound fluids, but there was no difference in the absolute protein carbonyl content in acute and chronic wound exudate. However, chronic wound fluids were found to have lower protein content, thus the normalized protein carbonyl content in chronic wound was found to be 15% higher [11]. This highlights serious methodological challenges associated with measurement of oxidative damage in wound fluids because its composition can vary considerably with the hydration state of the patient. These studies on wound fluids also do not answer the fundamental question of whether wounding induces oxidative damage.

Using thiobarbituric acid reactive substances (TBARS) as a biomarker of lipid peroxidation, early studies have actually found reduced lipid peroxidation in wounds compared to intact skin [12], [13]. However, it should be noted that measurement of TBARS is a poor marker of lipid peroxidation and is susceptible to artefacts [14]. Other authors have shown increases in oxidative damage between wounds from wild type and peroxiredoxins-VI deficient mouse models but the levels of oxidative damage in intact skin were not reported [9], [15].

In the present study, we have two main objectives. First, we aimed to measure the changes in oxidative damage over time in a full-thickness excision wound model. Second, we modulated the level of ROS by the topical application of H_2_O_2_ to determine if excessive oxidative damage could contribute to poor healing of wounds. Three biomarkers of oxidative damage, namely the F_2_-isoprostanes, protein carbonyls and 3-nitrotyrosine were used to determine changes in level of oxidative damage.

## Materials and Methods

### Materials

Radioimmunoprecipitation assay (RIPA) buffer was purchased from Cell Signaling Technology (Danvers, MA, USA). Rat Anti-MMP-8 (Cat.#: 2145-1) was purchased from Epitomics (Burlingame, CA, USA). Rabbit anti-CD31 (Cat#: ab28364) and rabbit anti-MMP-9 (Cat#: ab38898) were purchased from Abcam (Cambridge, UK). Rat monoclonal anti-mouse F4/80 and 7/4 antibodies were purchased from Serotec (Raleigh, NC, USA). Rat anti-TIMP-1 (Cat#: MAB980) was purchased from R&D systems (Minneapolis, MN USA). Prolong-gold anti-fade mount medium with DAPI was purchased from Life Technologies. Phosphatase inhibitor cocktail, PhosSTOP, and protease inhibitor cocktail, Complete mini-EDTA, were purchased from Roche (Basel, Switzerland). Vectorstain peroxidase Avidin biotin complex (ABC) kit was purchased from Vector Labs (Burlingame, CA, USA). The hematoxylin used was Shandon Instant Haematoxylin purchased from Thermofisher (Waltham, MA, USA). Horseradish peroxidase conjugated goat anti rabbit secondary antibody (Cat 0031460), horseradish peroxidase conjugated goat anti mouse secondary antibody (Cat 0031430), enhanced chemiluminescence substrate and N,O-bis(trimethylsilyl) trifluoroacetamide +1% trimethylchlorosilane (BSTFA+TMCS) silylating agent were obtained from Pierce Chemicals (Rockford, IL, USA). The oxyblot protein oxidation detection kit, 3-nitrotyrosine ELISA kit and suspension array ELISA kit for the detection of mouse CXCL1, CXCL5, CCL2 and CCL3 were purchased from Millipore (Billerica, MA, USA). All other chemicals were purchased from Sigma-aldrich (St. Louis, MO,USA).

### Animal Handling and Excision Wound Model

**Figure 1:**
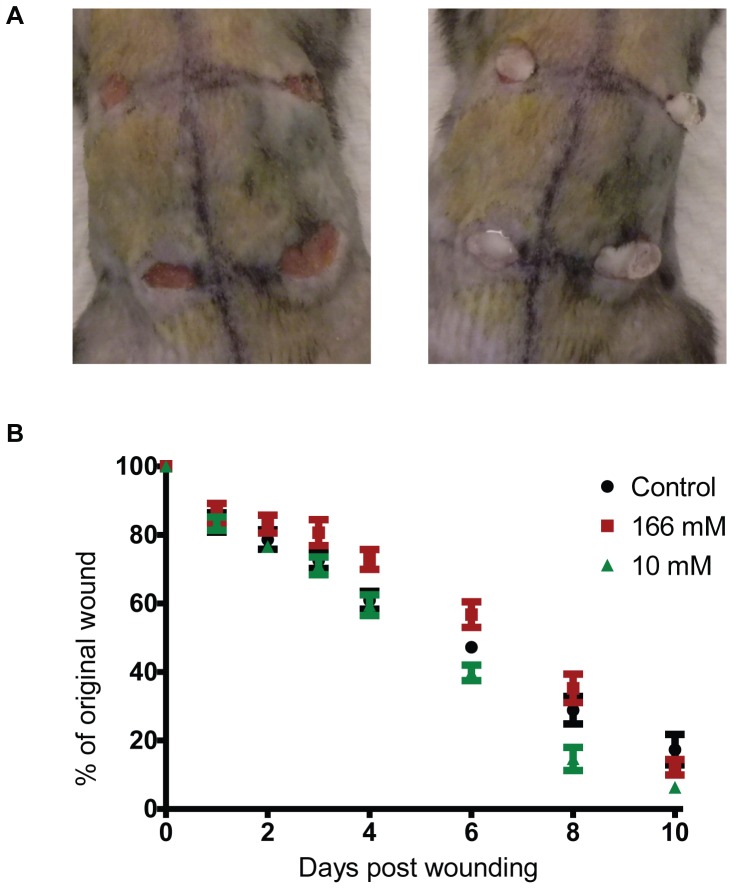
Low concentrations of H_2_O_2_ promoted wound closure but high concentrations of it delay wound closure. (A) Four full thickness excision wounds were created on each mouse and 15 µL of H_2_O_2_ was added into the wound cavity as shown. The left panel is a representative picture of the wounds generated. The right panel shows how the H_2_O_2_ is applied onto the wounds. (B) Effects of different concentrations of H_2_O_2_ on wound closure rate. The wound size of 6 to 8 mice was monitored for 6 and 10 days respectively before they were euthanized. The graph shown is the mean ± S.E.M. of the pooled results. 1-way ANOVA was used to analyze wound size. The differences between 166 mM H_2_O_2_ and control (p<0.05) or 10 mM H_2_O_2_ (p<0.01) were statistically significant on day 6. The differences between 10 mM H_2_O_2_ and control (p<0.05) were statistically significant on day 8 and 10. The differences between 10 mM and 166 mM H_2_O_2_ were also statistically significant on day 8 (p<0.05) but not on day 10.

Eight week old C57/BL6 mice were obtained from the NUS Laboratory Animal Care centre. The method for generating the excision wound and subsequent monitoring was approved by the NUS Institutional Animal Care and Use Committee (NUS 095/09). Animals were fed on a standard chow diet and housed in a specific pathogen free facility. Mice were acclimatized for a week before wounding. The procedure was carried out under anesthesia induced by isoflurane. Four full thickness excision wounds were created with a 5-mm dermal punch. The size of the wounds was then immediately traced onto a piece of sterile transparent plastic sheet. 15 µL of PBS or H_2_O_2_ diluted in PBS was then added carefully into the wound (Figure 1A). The mice were maintained under anesthesia for another 5 min to allow the H_2_O_2_ to be absorbed by the wound. An Elizabethan mouse collar was then placed on the neck to prevent the mice from biting or licking the wounds. Mice were also singly housed to prevent them from biting or licking each others’ wounds. 0.1 mg/kg of buprenorphine was administered subcutaneously immediately after wounding and also every 24 h for up to 4 days post wounding for pain relief.

For wound size monitoring, mice were anaesthetized and wound size was measured by tracing onto a piece of sterile transparent plastic sheet as described in the previous section. PBS or H_2_O_2_ were then reapplied onto the wound. For tissue collection, mice were euthanized by CO_2_ asphyxiation and wound tissues were removed using a 10 mm dermal punch. Tissues were processed depending on the assays that were to be carried out.

### Preparation of Histological Sections

Freshly excised wound samples were halved and processed for either paraffin or cryosectioning. Samples for paraffin sectioning were fixed immediately in 10% phosphate buffered formalin (pH 7.4) immediately after they were collected. Samples were routinely dehydrated, cleared and mounted in paraffin. Paraffin sections of 5 µm thickness were prepared on adhesive glass slides. Freshly collected wound samples for cryosections were embedded in optimal cutting temperature (OCT) compound and frozen using a dry ice and isopentane slurry. Cryosections of 6–8 µm thickness were prepared on adhesive glass slides.

### Masson’s Trichrome Stain

Paraffin sections were dewaxed, rehydrated and stained using the Masson-Goldner trichrome staining kit according to the manufacturer’s instruction. Photomicrographs were taken at 20X objective lens with bright field illumination using a Olympus BX 51 microscope. An image of the entire wound region was produced by piecing multiple fields together using Adobe Photoshop CS5. The number of green pixels in the neodermis was quantified and expressed as a fraction of the size of the wound region. The neodermis refers to the newly-formed granulation tissue beneath the hyper-proliferating epidermis and/or eschar that is devoid of hair follicles.

To quantify the number of green pixels, we developed a custom software for color image segmentation that was implemented as an ImageJ plug-in. The software can be downloaded for free at http://web.bii.a-star.edu.sg/archive/colseg/. A description of the software and a user manual is provided in the supplementary information (Manual S1).

### Immunohistochemical Stain of CD31

CD31 was stained using an immunohistochemical method. Paraffin sections were dewaxed and rehydrated. Sections were boiled in 10 mM pH 6.0 sodium citrate buffer for 15 min. Samples were then processed with a peroxidase ABC amplication kit with some modifications from the manufacturer’s protocol. Sections were blocked in 1.5% goat serum in tris-buffered saline (TBS) overnight at 4°C. After blocking, sections were incubated with rabbit polyclonal anti-CD31 diluted in 1.5% goat serum at a dilution of 1∶50 for 1 h. Slides were then washed in TBS before incubation with a biotinylated anti-rabbit secondary antibody at a dilution of 1∶200 for 1 h. Slides were then washed incubated with the avidin-biotin cassette reagent for 30 min. The peroxidase activity was stained by using diaminobenzidine using DAB+ according to the manufacturer’s protocol for 30 min followed by washing in TBS and counterstaining with hematoxylin. Slides were dehydrated, cleared and mounted with DPX mounting medium.

Photomicrographs of the sections were taken at 20X objective lens with bright field illumination using a Olympus BX 51 microscope. An image of the entire wound region was produced by combining multiple fields together using Adobe Photoshop CS5. The number of blood vessels within the neodermis was counted and normalized against the cross-sectional area of each section. Counting was done blinded by 2 lab members and results are average of both their counts. The average difference between the counts of each user from the mean is 14.8%.

### Immunofluorescence Staining

Frozen sections were fixed in 4% paraformaldehyde for 20 min. Sections were blocked with the Image-iT FX signal enhancer for 2 h. They were then incubated with the primary antibodies, anti-mouse F4/80 or anti-7/4, for 2 h at room temperature at 1∶1000 in TBS. The secondary antibody used was Alexa Fluor 594 goat anti-rat antibody at 1∶1000 dilution in TBS for 30 min at room temperature. Slides were mounted with anti-fade mounting medium containing DAPI and allowed to cure on a flat surface in the dark overnight. Slides were visualized and photographed using Olympus BX 51 microscope with consistent exposure time. An image of the entire neodermis was produced by combining multiple fields together using Adobe Photoshop CS5. Image analysis was carried out using ImageJ (NIH). Images were first split into RGB channels. Background correction was carried out using a rolling ball radius of 50 pixels. The fluorescence intensity of the neodermis was then measured. Negative controls with no primary antibody incubation showed no fluorescent staining.

### Protein Extraction

Wound tissues for protein analysis were frozen in dry ice immediately after collection and stored at −80°C until analysis. For protein extraction, two wound tissues (approximately 100 mg) from an animal were cut into small pieces and 500 µL of ice-cold RIPA buffer with protease and phosphatase inhibitor cocktail was added. The samples were ultrasonicated with a high intensity ultrasound probe (Sonics Vibra-Cell, Newtown, CT, USA) and then centrifuged for 10 min at 10 000 g, 4°C. The supernatant was collected and used for western blot, 3-nitrotyrosine ELISA, multiplex cytokine analysis and protein carbonyl assay.

### Western Blot Analysis

Thirty micrograms of protein were electrophoresed on a 10% SDS-polyacrylamide gel for the analysis of ERK1/2, p38 MAPK, MMP-8, MMP-9 and TIMP-1. Gels were wet-transferred onto nitrocellulose membranes. Membranes were blocked with 5% skim milk in TBST for 1 h at room temperature prior to incubation with the antibodies overnight at 4°C. Dilutions for all antibodies were 1∶1000 except for p-ERK1/2 which was diluted 1∶2000. Blots were visualized by chemiluminescence using a Chemidoc XRS imaging system (Bio-Rad, Milan, Italy). Densitometry was carried out using ImageJ.

### 3-Nitrotyrosine ELISA

Analysis was carried out according to the manufacturer’s instruction. 300 µg of protein was loaded per well. A standard curve was plotted in GraphPad Prism by fitting to the five-parameter logistic equation. Levels of 3-nitrotyrosine in samples were determined using the interpolation function in the software.

### Multiplex Cytokine Analysis

Analysis was carried out according to the manufacturer’s instructions. 75 µg of protein was loaded per well. Analysis was carried out on a Millipore Milliplex system and results were analyzed using the Milliplex analyst software.

### F_2_-Isoprostanes Extraction and Analysis

Wound tissues for F_2_-isoprostane analysis were frozen in dry ice immediately after collection and stored at -80°C until analysis. F_2_-isoprostanes were analysed using previously published methods with slight modification [16], [17]. Lipids were extracted from 2 whole wounds (approximately 100 mg). The wounds were homogenized in 0.5 ml PBS (pH 7.4) and 1 ml Folch organic solvent mixture (CHCl_3_:methanol, 2∶1 v/v, +0.005% BHT) at 4°C. After centrifugation at 2300 *g* for 10 min the lower organic layer was carefully transferred to a glass vial and dried under a stream of N_2_. The dried lipid extract was resuspended in 0.25 ml deionized water and 0.25 ml of 1 M KOH (in pure methanol) was added to hydrolyse the lipids. Heavy isotopic F_2_-isoprostane and arachidonic acid internal standards (0.5 ng IPF_2a_- VI-d_4_, 0.25 ng 8-iso-PGF_2a_-d_4_, 0.5 ng IPF_2a_-IV-d_4_ and 1.0 µg arachidonic acid-d_8_ in 20 µl ethanol) were also added.

N_2_ gas was introduced into each sample vial, which was then capped to prevent any further oxidation. Hydrolysis was done overnight at ambient temperature in the dark. 1 ml of deionised water and 1 ml of 40 mM acetic acid were added after the hydrolysis. The pH of samples was adjusted to 4.5 using 6 M HCl.

60 mg MAX (mixed ion exchange, Waters) SPE columns were preconditioned with 2 ml of methanol followed by 2 ml of 40 mM formic acid (pH 4.5). The lipid extract was then loaded into the column and the column was rinsed with 2 ml of methanol:20 mM formic acid, pH 4.0 (3∶7). 2 ml hexane followed by another 2 ml of acetone:hexane (3∶7) were introduced into the column. Finally, arachidonic acid and F_2_-isoprostanes were eluted from the SPE column with 1.8 ml of acetone:methanol (4∶1), collected and dried under N_2_ gas.

Samples were derivatized with 12.5 µl of DIPEA (10% v/v acetonitrile) and 25 µl of PFBBr (10% v/v acetonitrile) at room temperature for 30 min and dried under nitrogen gas. To the dried samples, 12.5 µl of acetonitrile and 25 µl of BSTFA + TMCS were added and the reaction mixtures incubated at room temperature for 1 h silylation and then dried. The derivatized samples were reconstituted in 30 µl of iso-octane and incubated at room temperature for 2 min before GC-MS analysis.

Derivatized samples were analyzed by a Hewlett–Packard 5973N mass selective (MS) detector (Agilent Technologies) interfaced with a Hewlett–Packard 6890 gas chromatograph (GC) (Agilent Technologies), fitted with an automatic sampler and a computer workstation. The injection port and GC-MS interface were kept at 270 and 300°C, respectively. The mass spectrometer was used in the negative chemical ionization (NCI) mode with the ion source and quadrupole at 150 and 106°C, respectively, and the methane flow rate was set to 2 ml/min. Chromatographic separations were carried out on a fused silica capillary column (30 m × 0.2 mm i.d.) coated with cross-linked 5% phenylmethylsiloxane (film thickness 0.33 µm) (Agilent Technologies). The carrier gas, helium, was set to a flow rate of 1 ml/min. Derivatized samples (1 µl) were injected splitless into the GC injection port. The column temperature was maintained at 180°C for 0.75 min, then increased to 275°C at 40°C/min, and then held at 275°C for 9 min. Finally the temperature was raised to 300°C at 40°C/min and held for 10 min. Selected ion monitoring was performed to monitor the carboxylate ion (M-181: loss of pentafluorobenzyl, CH_2_C_6_F_5_) at ions *m/z* 569 for 8-iso-PGF_2α_ and at *m/z* 573 for deuterium-labeled (8-iso-PGF_2α_-d_4_ and IPF_2α_-VI-d_4_) internal standards. Quantitation was achieved by relating the peak area of the total F_2_-IsoPs with the sum of the internal standard peaks of the two different F_2_-IsoPs (8-iso-PGF_2α_-d_4_ and IPF_2α_-VI-d_4_). Reported values are the sum of 8-iso-PGF_2α_, IPF_2α_-VI and IPF_2α_-IV.

Arachidonate was also analyzed by GC-MS-NCI using the same instrument and column conditions as described above, except for the following: helium was set to a flow rate of 1 ml/min and derivatized samples (1 µl) were injected splitless into the GC injection port. The column temperature was maintained at 180°C for 0.75 min, then increased to 310°C at 40°C/min, and then held for 8 min. Target and qualifier ions were chosen for selected ion monitoring mode of the GC-MS to monitor the carboxylate ion (M-181: loss of pentafluorobenzyl, CH_2_C_6_F_5_) at ions m/z 303 for arachidonate and m/z 311 for deuterium-labeled arachidonic acid-d8 internal standard. Quantitation was achieved by relating the peak area of the total arachidonate with the internal standard peak. The retention times for 8-iso-PGF_2α_, IPF_2α_-VI, IPF_2α_-IV and arachidonic acid were 11.3, 11.3, 11.50 and 5.2 min respectively.

### Protein Carbonyl determination

Protein carbonyls were determined using the OxyBlot protein oxidation kit. After tissue homogenization, 25 µl of tissue lysate was mixed with 10 µl of 12% SDS and 20 µl of 20 mM 2,4-dinitrophenylhydrazine (DNPH). After 15 min of incubation at room temperature, 15 µl of neutralization solution (2 M Tris, 30% glycerol) was added. The volume equivalent to 3.8 µg of protein was then loaded into a slot blot apparatus (Bio-Rad, Hercules, USA) and transferred onto a nitrocellulose membrane under vacuum. The membranes were blocked with 5% skim milk in TBST for 1 h before probing with anti-DNPH antibody (1∶150) and HRP conjugated anti-rabbit IgG antibody (1∶300) for 1 h each. Blots were visualized by chemiluminescence using a Chemidoc XRS imaging system.

## Results

### 10 mM H_2_O_2_ Promoted Wound Closure but 166 mM Retarded it

Topical application of H_2_O_2_ at 166 mM was found to delay wound healing (Figure 1B) when compared to the control mice. This concentration will deliver 2.5 µmole of H_2_O_2_ per wound. Pharmacologically, this concentration is also equivalent to a 0.5% solution of H_2_O_2_, which is similar to the concentrations commonly used for disinfection (0.5 to 3%). Even though H_2_O_2_ at 166 mM delayed wound closure initially, the wound closure rate accelerated during the latter part of the healing process and there were no differences in wound size by day 8 and day 10 when compared to control mice. H_2_O_2_ at 10 mM increased the wound closure rate slightly (Figure 1B) compared to control mice.

It appears that topical application of H_2_O_2_ at 166 mM delayed the initial healing process in mice. We therefore chose to examine the healing process in detail on day 6 post-wounding, which is mid-way through the healing process. Connective tissue formation was measured using the Masson’s trichrome stain [18]. The Masson’s trichrome stain differentially color connective tissues, predominantly collagen, as they are less porous compared to the other tissue components. Angiogenesis was evaluated using an immunohistochemical stain of CD31, a cell surface marker of endothelial cells.

**Figure 2:**
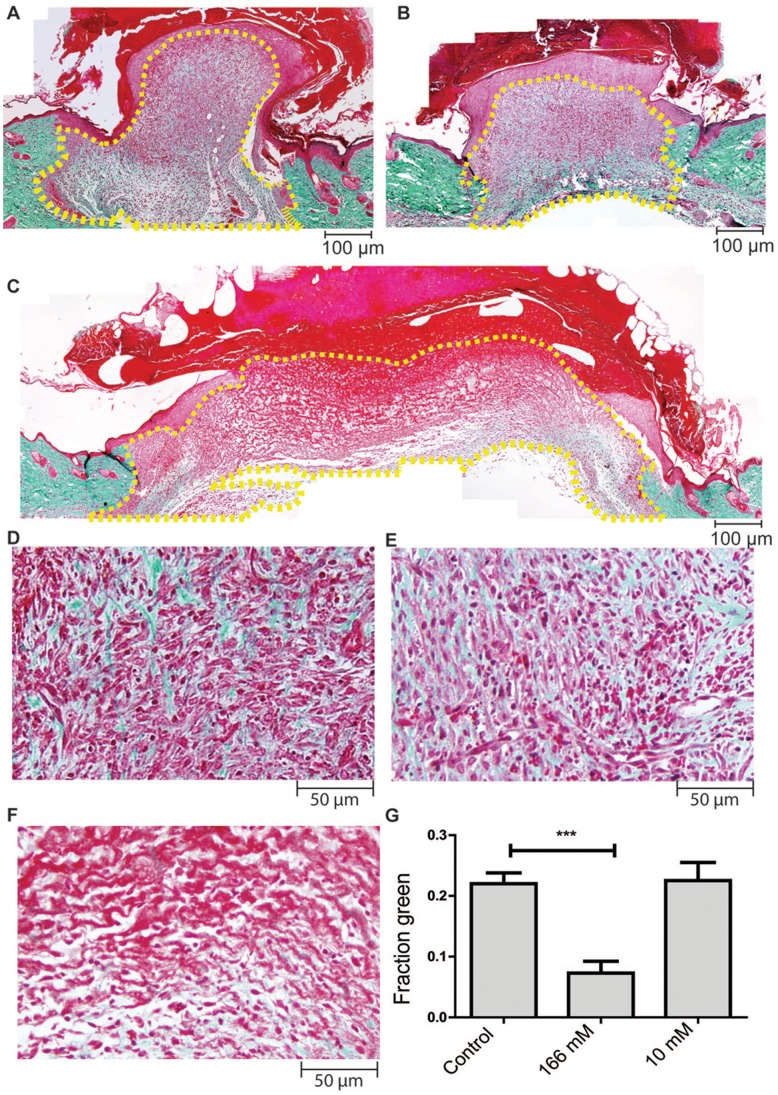
High concentrations of H_2_O_2_ retard connective tissue formation. Paraffin sections from day 6 wounds were stained with the Masson-Goldner trichrome stain as described in material and method. Connective tissues are stained green. Fibrin, eschar and cytoplasm are stained red. Nuclei are stained dark brown. Representative images for control (A,D) 10 mM (B, E) and 166 mM (C, F) treated wounds are shown. Images A-C are at 100X magnification while D-F are at 200X magnification. (G) Quantification of the fraction of pixels that are stained green. The number of pixels stained green within the neodermis at 100X magnification was quantified using a custom software. The area quantified is outlined with the dashed line. Results were analyzed using 1-way ANOVA followed by Dunnett’s multiple comparison test with control. Graph shown is the mean ± S.E.M. n = 6–7, *** p<0.001.

**Figure 3:**
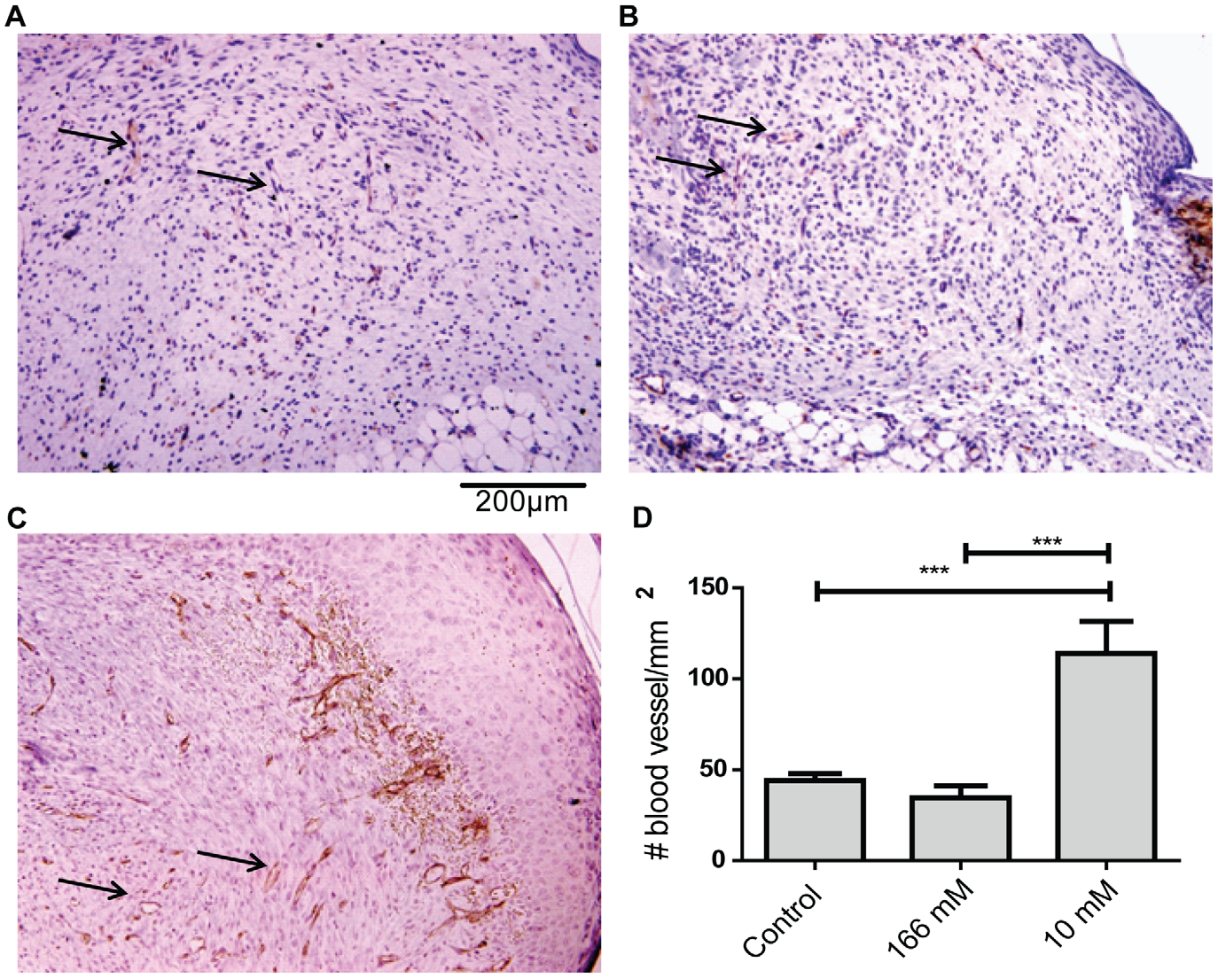
Low concentrations of H_2_O_2_ increased wound angiogenesis. Paraffin sections from day 6 wounds were stained for CD31 using an immunohistochemical method. Representative photomicrograph of (A) control, (B) 166 mM H_2_O_2_ and (C) 10 mM H_2_O_2_ treated wounds are shown. (D) The number of brown lumen-like structures within the neodermis was counted in a single blinded fashion and analyzed using 1-way ANOVA followed by Dunnett’s multiple comparison test with control. Graph shown is the mean ± S.E.M, n = 6–7, *** p<0.001.

Wounds treated with 166 mM H_2_O_2_ showed decreased connective tissue formation as signified by reduced amount of area stained green compared to controls. 10 mM H_2_O_2_ did not affect connective tissue formation (Figure 2). 166 mM H_2_O_2_ did not affect angiogenesis but 10 mM H_2_O_2_ strongly promoted it (Figure 3). These results from the connective tissue and CD31 immunostaining corresponded with our observation that 166 mM H_2_O_2_ delayed wound closure but 10 mM H_2_O_2_ promoted it.

### H_2_O_2_ Increases Levels of MMP-8 in Wounds

**Figure 4:**
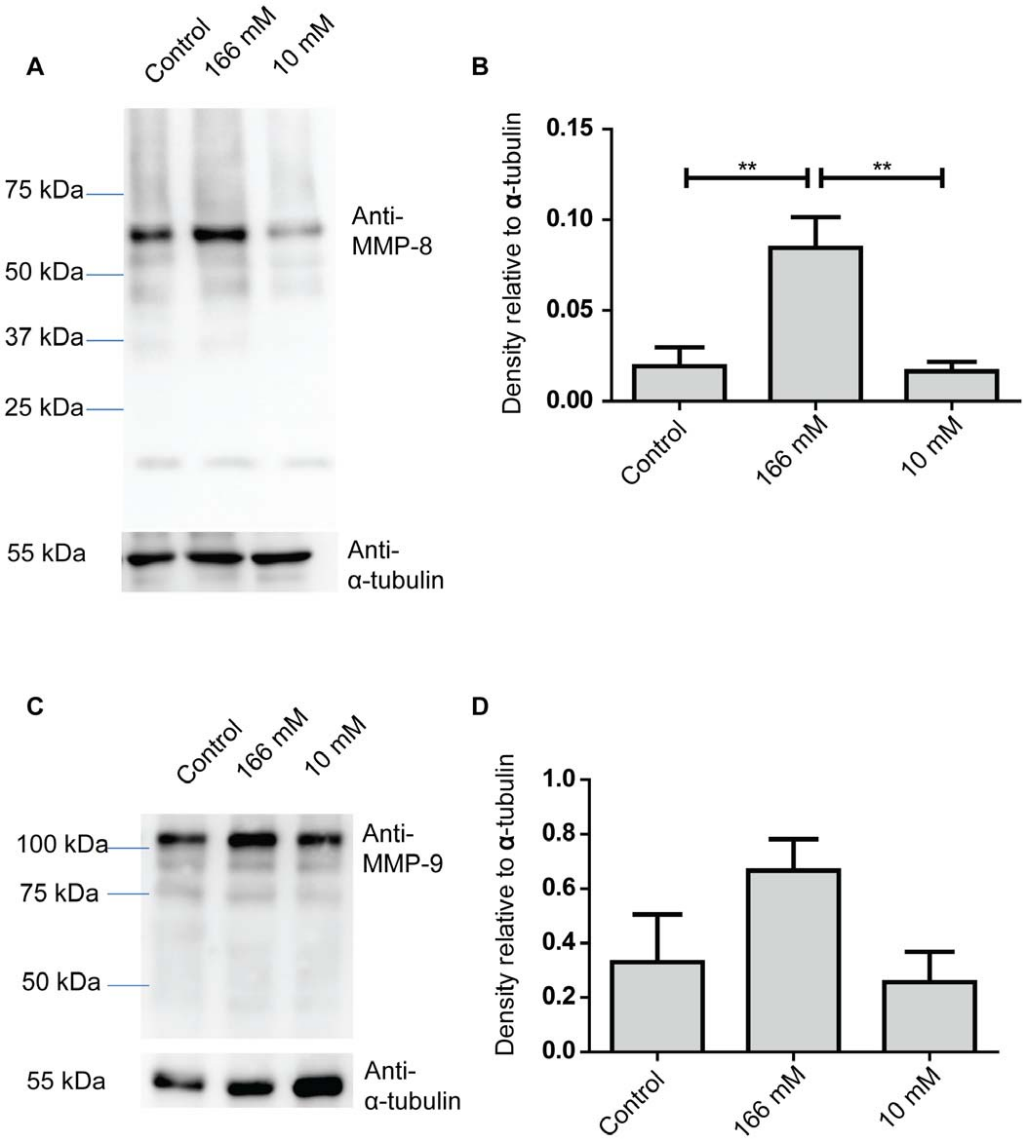
High concentrations of H_2_O_2_ increase levels of MMP-8 in wounds. Western blot analysis of wound tissues lysate collected 6 days after wounding. Each lane represents a sample from a different animal. (A) Representative blot of MMP-8. (B) Densitometry analysis of MMP-8 normalized against α-tubulin re-probed from their respective blot. Results are mean ± S.E.M. (n = 4) and were analyzed using 1-way ANOVA followed by Tukey multiple comparison among all the columns. ** p<0.01 (C) Representative blot of MMP-9. (D) Densitometry analysis of MMP-9 normalized against α-tubulin re-probed from their respective blot. Results are mean ± S.E.M. (n = 4), p values for 1-way ANOVA is p = 0.13.

Increased MMP-8 and 9 have both been observed in chronic wounds [26]. Therefore we hypothesized that H_2_O_2_ might increase levels of MMP-8 and 9 in wounds and possibly lead to reduced connective tissue deposition. Levels of MMP-8 and 9 in wounds were measured using Western blots (Figure 4).

Western blots of MMP-8 had a strongly immunoreactive band at about 57 kDa, which corresponds to the activated form of MMP-8 derived from polymorphonuclear cells. Weak immunoreactivity was also observed at 21 kDa, which has been previously reported to be a degraded fragment of MMP-8 [19]. Weak immunoreactivity was also observed at 45 and 55 kDa bands which could be MMP-8 derived from non- polymorphonuclear cells [20].

Densitometry result analysis of the 57 kDa band is shown in Figure 4B. It was found that topical application of 166 mM H_2_O_2_ increased the protein levels of the activated MMP-8 in wounds but 10 mM did not affect the protein levels of MMP-8 (p<0.01). Densitometry analysis was also carried out for the 21 kDa fragment and it was found to follow the same trend as the immunoreactive band at 57 kDa indicating that the degree of degradation is proportionate to amount of MMP-8 (data not shown).

Murine pro-MMP-9 appears as a band 105 kDa in size on SDS-PAGE while activated MMP-9 appears as 97 kDa in size [21]. We observed bands with strong immunoreactivity with molecular weight that correspond to the zymogen but not the cleaved active forms. H_2_O_2_ was found to increase levels of pro-MMP-9 but the increase was not statistically significant. *p*-value for one-way ANOVA was 0.1329.

The effect of H_2_O_2_ on the levels of tissue inhibitor of metalloproteinase 1 (TIMP-1) was also measured (Figure S1). Two bands were observed at approximately 28 kDa which could be due to different glycosylated isoforms [22]. From densitometry analysis, H_2_O_2_ was not found to cause any change in the levels of TIMP-1, regardless of the concentration used.

### High Concentrations of H_2_O_2_ Increase Neutrophil Infiltration

**Figure 5:**
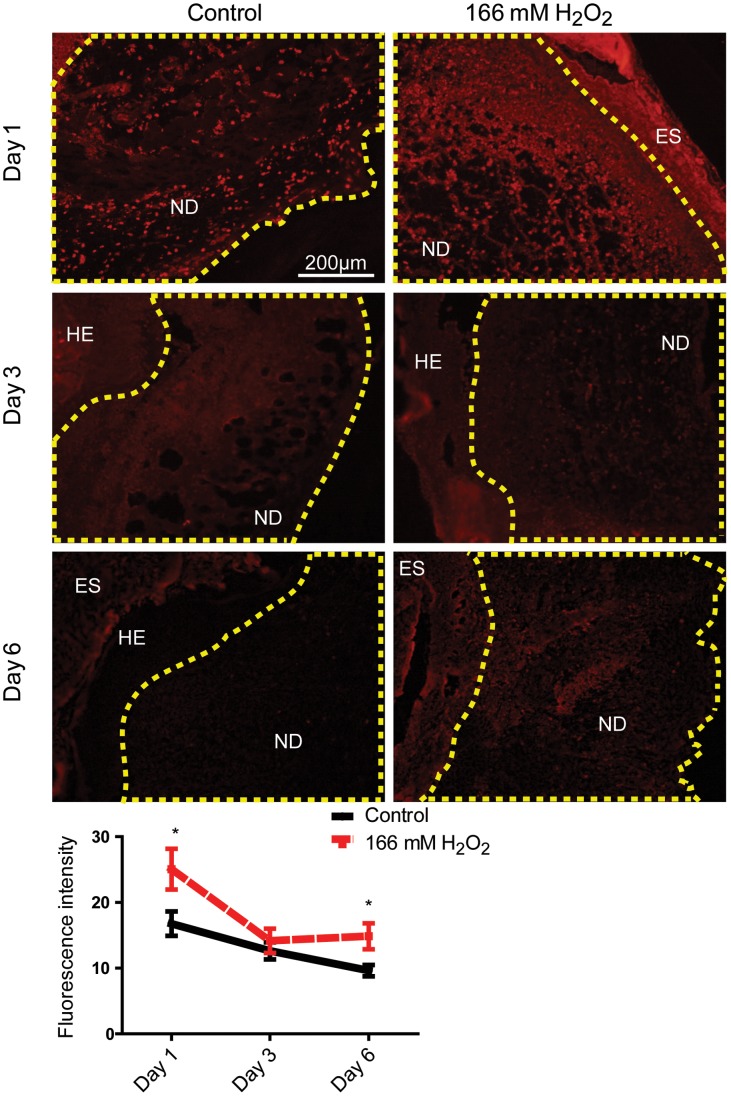
166 mM H_2_O_2_ increased neutrophil infiltration in day 1 and 6 wounds. Fluorescence intensity of the neodermis was quantified using ImageJ. The area quantified is outlined with the dashed line. Results shown are mean ± S.E.M, n = 6-7. A representative section from each treatment is shown. ES – Eschar; HE – Hyper-proliferating epidermis; ND – neodermis.*p<0.05.

The effect of H_2_O_2_ on neutrophil and macrophage infiltration was also evaluated using immunofluorescence staining (Figure 5). In control mice, we observed strong immunofluorescence staining of neutrophils day 1 post wounding which declined as the wound healed, which was similar to trends reported in the literature [23]. However, wounds treated with 166 mM H_2_O_2_ showed stronger immunofluorescence staining on day 1 and 6 when compared to control wounds. This indicates that H_2_O_2_ caused a larger infiltration of neutrophils which also persisted for a longer period of time. On the other hand, there was no change in neutrophil infiltration in wounds treated with 10 mM H_2_O_2_ (Figure S2). Both concentrations of H_2_O_2_ were not found to affect macrophage infiltration (Figure S3).

### High Concentrations of H_2_O_2_ Increase ERK1/2 and p38 Phosphorylation

**Figure 6:**
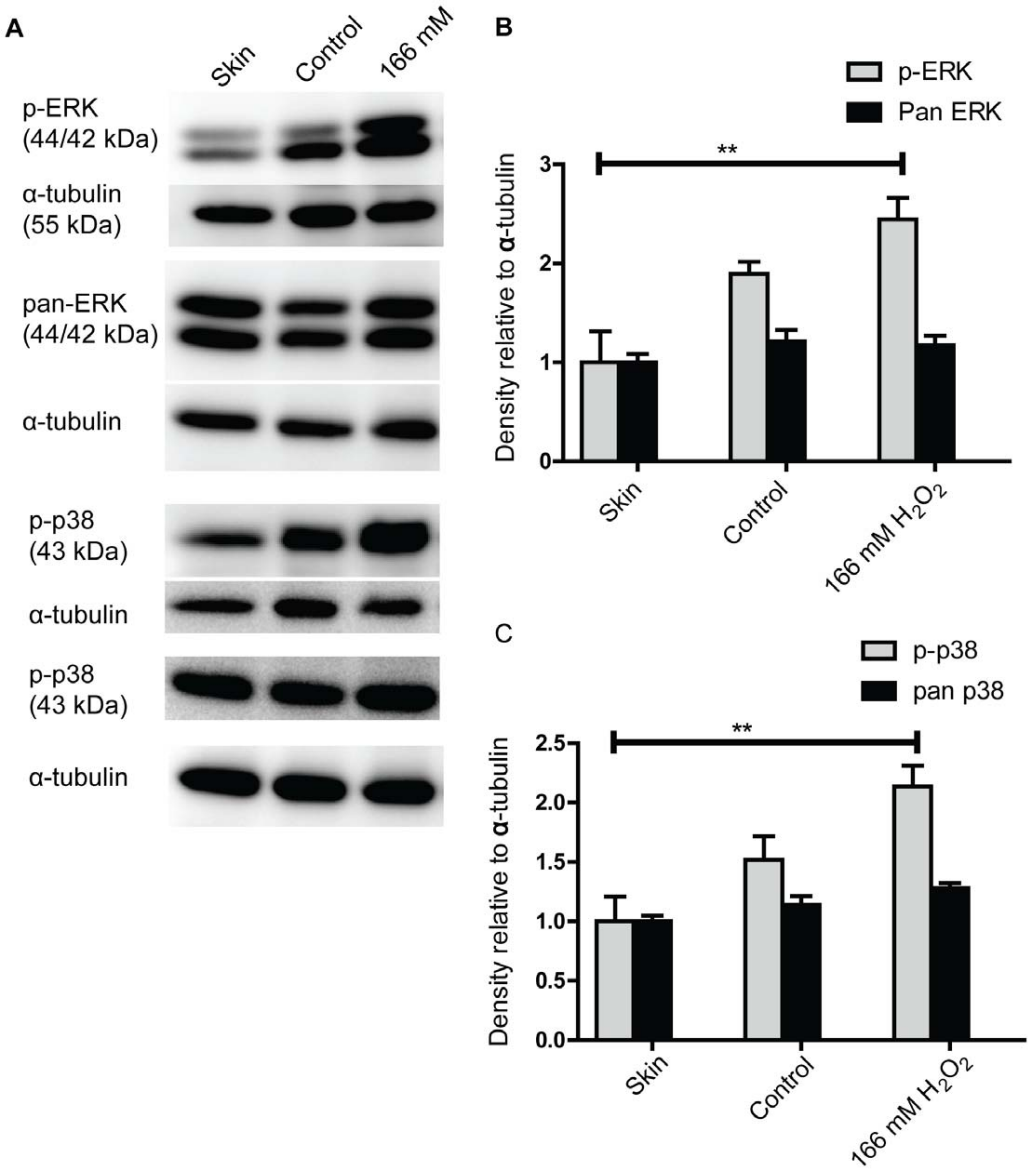
Wounding increases ERK1/2 and p38 phosphorylation which can be further increased by 166 mM H_2_O_2_ treatment. (A) Representative blots of wound tissues lysate collected 30 min after wounding. Skin denotes skin from non-wounded animals while control refers to wounds treated with PBS. (B) The density of phosphorylated ERK and pan-ERK and (C) phosphorylated p38 and pan p38 were normalized against α-tubulin re-probed from their respective blot. Results shown are mean ± S.E.M. (n = 4). Densitometry results were analyzed by 1-way ANOVA and test of significance between all column was determined using Tukey’s post hoc test. Only the comparison between 166 mM treated wounds and skin was statistically significant for both B and C. ** p<0.01.

We have previously found that H_2_O_2_ might improve wound healing by activating the MAPK pathway in a keratinocyte scratch wound model [24]. We therefore proceeded to investigate if H_2_O_2_ also has an effect on the MAPK pathway in this *in vivo* model of wound healing. Western blot analysis comparing intact skin with wound edge tissues showed that wounding activates both ERK1/2 and p38 signaling and an increase in phosphorylation was observed 30 min after wounding. (Figure 6). It was also observed that treatment with 166 mM H_2_O_2_ increases the level of phosphorylation further. However, the phosphorylation signal had attenuated by 4 h after wounding and no difference in phosphorylation was observed between skin, control wounds and H_2_O_2_ treated wounds (Figure S4). This is in contrast to the persistent (at least 8 h) ERK1/2 and p38 signal that we observed in keratinocytes treated with H_2_O_2_ in cell culture [24].

### 166 mM H_2_O_2_ Delay Healing without Increasing Oxidative Damage

**Figure 7:**
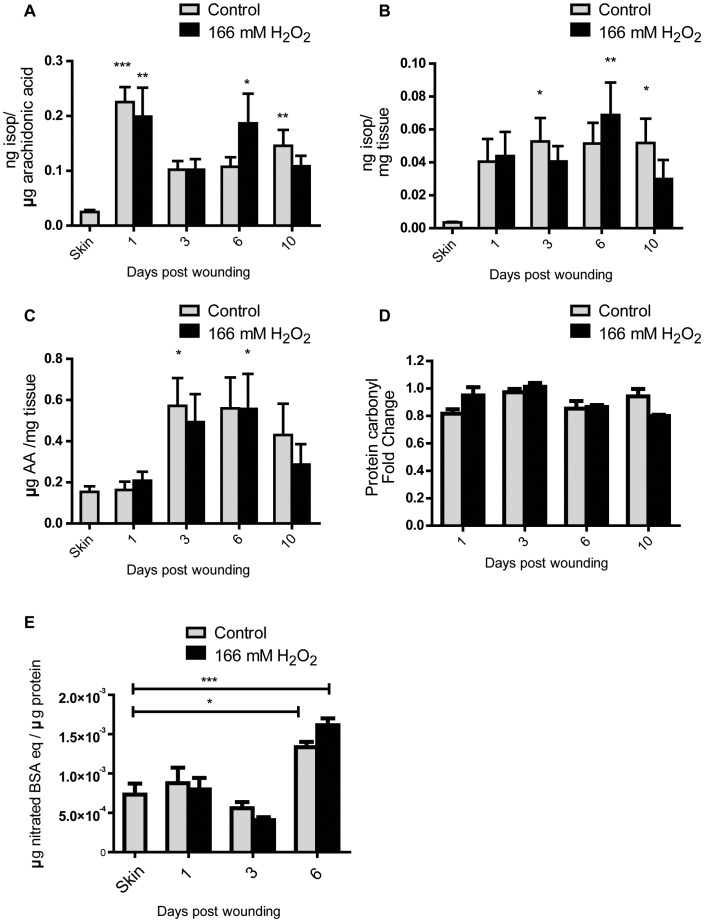
Wounding increased lipid peroxidation and nitrative damage but not protein carbonylation. Levels of F_2_-isoprostanes levels in skin and wounds were compared by normalizing against arachidonic acid (A) or tissue weight (B). Results shown are mean ± S.E.M, n = 5. Wounds were compared to skin using 1-way ANOVA with Dunnett’s post-hoc test. Asterisks denote level of significance when compared to skin. Control and H_2_O_2_ wounds were also compared against each other using 2-way ANOVA but the differences was not statistically significant. (C) Levels of arachidonic acid in skin and wound tissues. Results shown are mean ± S.E.M, n = 5. Wounds were compared to skin using 1-way ANOVA with Dunnett’s post-hoc test. Asterisks denote level of significance when compared to skin. Control and H_2_O_2_ wounds were also compared against each other using 2-way ANOVA but the differences was not statistically significant. (D) Levels of protein carbonyls in wounds were compared to intact skin and expressed as fold change. The results shown are the mean fold change ± S.E.M. No difference in the levels of protein carbonyl was observed in control wounds and 166 mM H_2_O_2_ treated wounds. (E) Comparison of 3-nitrotyrosine level in skin and wounds. Results shown are mean ± S.E.M., n = 5. The 3-nitrotyrosine levels of skin were compared to control wounds or H_2_O_2_ treated wounds and analyzed with 1-way ANOVA followed by Dunnett’s post-hoc test. Levels of 3-nitrotyrosine were significantly higher at day 6 after wounding. Levels of 3-nitrotyrosine in control and 166 mM H_2_O_2_ treated wounds were also compared using 2-way ANOVA and the differences were not statistically significant. *p<0.05, ** p<0.01, ***p<0.001.

From our results, it appears that 166 mM H_2_O_2_ delayed wound healing by creating a more proteolytic environment. However there is little information on whether H_2_O_2_ at high concentrations, such as at 166 mM, can induce oxidative damage in wounds. The levels of F_2_-isoprostanes in wounds were used as a robust indicator of lipid peroxidation [25]. Levels of F_2_-isoprostanes are usually normalized against arachidonic acid, the precursor fatty acid of the isoprostanes. However such normalization can be controversial [26], therefore we chose to show the levels of F_2_-isoprostanes with and without normalization against arachidonic acid. The total levels of type III, IV and VI isoprostanes were summed up and expressed as per unit arachidonic acid (Figure 7A) and per unit tissue weight (Figure 7B).

As shown in Figure 7A, wounding strongly increased levels of F_2_-isoprostanes at 1 day after wounding for both control and H_2_O_2_ treated wounds. The increases in F_2_-isoprostanes in day 6 H_2_O_2_ and day 10 control wounds were also statistically significant when compared to intact skin. While the increases in F_2_-isoprostanes for the other time points were not statistically significantly, it should be noted that their 95% confidence intervals (not shown) do not overlap with that of intact skin. Therefore, the increase in F_2_-isoprostanes on day 3 and 6 may still be biologically important. The levels of F_2_-isoprostanes in control and H_2_O_2_ treated wounds were also compared using 2-way ANOVA but the differences were not statistically significant. This indicates that the H_2_O_2_ applied did not induce additional lipid peroxidation.

We also showed the level of F_2_-isoprostanes normalized against the tissue weight instead of arachidonic acid. The results showed a similar trend to the levels of F_2_-isoprostanes normalized against arachidonic acid but with a larger standard error for all data points (Figure 7B). This implies that normalizing against arachidonic acid might be important to account for variations in the tissues. Interestingly, we also found that the amount of arachidonic acid was higher in wounds compared to intact skin, with the difference being statistically significant between intact skin, day 3 control wounds and day 6 H_2_O_2_ treated wounds (Figure 7C). This raises the possibility that the increase in F_2_-isoprostanes might be confounded by the increase in its precursor. However, it should be noted that the increase in arachidonic acid (increased from day 3) lags behind the increase in F_2_-isoprostanes (increased from day 1). Therefore we believe that the increase in F_2_-isoprostanes cannot be simply due to increased levels of initial substrate. We also compared the levels of arachidonic acid between control and H_2_O_2_ treated wounds using 2-way ANOVA and found no significant differences.

ROS can also cause protein oxidation which can be assessed by measuring the levels of protein carbonyls present [27]. A slot blot method for measuring protein carbonyls was used and the densities of bands from wounds were compared to that of intact skin and expressed as fold change (Figure 7D). It was found that the levels of protein carbonyls present in wounds were comparable to the level in intact skin. 166 mM H_2_O_2_ also did not cause any further increase in the levels of protein carbonyls compared to control wounds.

We also assessed the level of reactive nitrogen species in wounds by measuring levels of 3-nitrotyrosine, a biomarker of nitrative damage [28]. Wounding was also found to increase levels of 3-nitrotyrosine (Figure 7E). In contrast to the F_2_-isoprostanes, levels of 3-nitrotyrosine was found to increase maximally on day 6 instead of day 1. H_2_O_2_ was also not found to increase nitrative damage.

## Discussion

It has been previously reported that topical application of 50 mM H_2_O_2_ can promote wound closure in a murine model of wound healing concomitant with increased angiogenesis. However 3% H_2_O_2_ (980 mM) was found to delay healing [3]. 3% H_2_O_2_ has also been shown to delay healing in a porcine model of wound healing together with a reduction in dermal thickness [29].

In our results, 10 mM H_2_O_2_ promoted wound closure and angiogenesis whereas 166 mM H_2_O_2_ (0.5%) caused a reduction in connective tissue formation and retarded wound closure. However, previous studies did not investigate the effect of H_2_O_2_ on the level of oxidative damage. In our study, we found that high concentrations of H_2_O_2_ retarded wound healing without increasing oxidative and nitrative damage. This implies that H_2_O_2_ can cause poor healing by other mechanisms besides causing oxidation of these biological substrates.

We found that a possible mechanism by which H_2_O_2_ could delay healing is by reducing connective tissue formation, possibly by increasing levels of MMPs. It has been postulated that excessive proteolysis could be a cause of poor wound healing in chronic wounds [30], [31]. In our study, we observed a statistically significant increase in MMP-8 levels and a smaller, non-significant increase in MMP-9 for wounds treated with H_2_O_2_. MMP-8 is the predominant collagenase in acute wounds and chronic wounds [32] while MMP-9 is the most abundant gelatinase in chronic wounds [31]. MMP-8 cleaves triple helical collagen at specific sites, leading to the auto-denaturation of collagen to gelatin which can be further broken down by gelatinases such as MMP-9 [33], [34]. As expected, overexpression of MMP-8 or -9 leads to poor healing [35], [36]. However, depleting MMP-8 or -9 has also been shown to delay healing [37], [38]. It appears that MMP-8 and -9 need to be present at an optimal level for the right length of time in order to achieve efficient healing, and that topical application of H_2_O_2_ may possibly disrupt the physiological balance.

TIMP-1 forms a 1∶1 complex with metalloproteinases to inhibit their activity [39]. It has been observed that chronic wounds tend to have lower levels of TIMP-1 and that this could be a cause for increased proteolysis in chronic wounds [31]. However we observed no differences in levels of TIMP-1 between wounds treated with different concentrations of H_2_O_2_ in our model.

There are several possible causes for the increased neutrophil infiltration observed in wounds treated with 166 mM H_2_O_2_. H_2_O_2_ could act as a chemoattractant for neutrophils. Wounding in zebrafish has been shown to induce H_2_O_2_ production, which in turn attracts neutrophils [1]. We have shown that H_2_O_2_ increases ERK1/2 phosphorylation, which has also been recently shown to be important in H_2_O_2_ -induced neutrophil chemotaxis [40]. Thus, it seems plausible that repeated application of a neutrophil chemoattractant on the wounds would result in increased inflammation.

Another possible mechanism for increased neutrophil infiltration is the release of damage-associated molecular pattern (DAMP) molecules. Injured cells can release diverse molecules, such as the nuclear protein HMGB1 and mitochondrial DNA, which not only attract neutrophils but also activate them [41]. H_2_O_2_ has been shown to induce the release of these danger signals by macrophages and monocytes in *in vitro* models [42].

It is also possible that H_2_O_2_ may increase the production of chemokines which might attract neutrophils. We have investigated this possibility by measuring CXCL1 (KC), CXCL5 (LIX), CCL2 (MCP-1) and CCL3 (MIP-1α) using a multiplex ELISA method but found that while wounding strongly increases the production of these chemokines, H_2_O_2_ does not alter the secretion profiles (Figure S5). However, a drawback of this method is that the bioactivities of these chemokines cannot be determined. Interestingly, MMP-8 has also been shown to cleave CXCL-8 and its mouse homologue CXCL5 and the resulting fragments shows stronger neutrophil chemotatic properties [43]. This could be yet another possible mechanism for the increased neutrophil infiltration we observed in H_2_O_2_ treated wounds.

Excessive neutrophil infiltration can cause tissue damage by producing ROS and various proteases. Therefore it has been suggested that excessive neutrophil infiltration may be a cause of poor wound healing [44]. However, the problem appears to be complex. For example it has been shown that there is persistent neutrophil infiltration in wounds of diabetic mice lacking leptin. Systemic administration of leptin improves healing and reduces neutrophil but not macrophage infiltration in these mice [45]. On the other hand, topical application of GM-CSF improved wound closure and neovascularization in mice with streptozocin-induced diabetes, a model of type I diabetes. However this improved healing was also associated with increased neutrophil and macrophage infiltration [46]. Depleting neutrophils has been variably shown to accelerate [47] or delay healing [48] by different groups. Perhaps the effects of sustained neutrophil infiltration vary between different models of wound healing.

Interestingly, although we observed increased neutrophil infiltration, we did not observe increased oxidative damage in terms of lipid peroxidation and protein nitration. This implies that the neutrophils may not have produced excessive ROS or the increase in ROS was effectively ameliorated by the endogenous antioxidant defence system. On the other hand, we observed increased levels of MMP-8, which is predominantly produced by neutrophils [19].

We have previously demonstrated that H_2_O_2_ induces persistent ERK1/2 and p38 phosphorylation in a keratinocyte cell culture model [24]. We further showed that persistent ERK1/2 phosphorylation is needed for its proliferative effects. However, we found that H_2_O_2_ did not induce persistent ERK1/2 and p38 phosphorylation in our *in vivo* wounds. It appears that exogenously applied H_2_O_2_ has different effects on cells in *in vivo* and *in vitro* conditions.

This is the first study, to the best of our knowledge, which systematically monitors changes in levels of oxidative damage over the course of healing. Levels of lipid peroxidation were found to be maximal 1 day after wounding but decreased and stabilized throughout the rest of the healing. The F_2_-isoprostanes were found to be excellent markers of oxidative damage in wound healing. Their levels were low but detectable in non-wounded skin but increased by 9-fold at 1 day after wounding. It was also observed that the levels of arachidonic acid also increase as a wound heals. This is consistent with reports of higher levels of arachidonic acid along with other polyunsaturated fatty acids in hypertrophic scars compared to normal skin [49]. These changes could be due to changes in the fatty acid biosynthesis in the keratinocytes and fibroblasts in response to inflammation and proliferation signals. They could also be due to changes in cellular composition of the wounds as inflammatory cells infiltrate the wounds, and different cell types may be differentially sensitive to H_2_O_2_ [50]. Further studies would be needed to determine the reason for change in fatty acid composition during wound healing.

The levels of nitrative damage were highest on day 6 after wounding with a 2-fold increase. On the other hand, we were unable to detect any changes in protein carbonyl. It appears that the F_2_-isoprostanes are more responsive and sensitive biomarkers for oxidative damage than the oxidized protein markers hence we would recommend F_2_-isoprostanes to be used in future wound healing studies.

In agreement with previous studies, we found that wounds display a biphasic response to topical application H_2_O_2_ where lower concentrations of it promoted healing while higher concentrations of it delays healing. However the delay in healing is not associated with increased in lipid peroxidation, protein oxidation or nitrative stress as measured by F_2_-isoprostanes, protein carbonyls and 3-nitrotyrosines.

## Supporting Information

Figure S1 **H_2_O_2_ did not affect levels of TIMP-1 in wounds.** Day 6 wound tissues were lysed and the amount of TIMP-1 was measured using western blot. (A) Representative blot of TIMP-1. (B) Densitometry analysis of both bands normalized against α-tubulin. The results shown are mean ± S.E.M. (n = 4). Results were analyzed using 1-way ANOVA and the differences were not significant. (p = 0.35)
(TIF)

Figure S2 **166 mM H_2_O_2_ increased neutrophil infiltration but 10 mM H_2_O_2_ does not.** Results shown are mean ± S.E.M, n = 6–7. A representative section from each treatment is shown. ES – Eschar; HE – Hyper-proliferating epidermis; ND – neodermis.*p<0.05
(TIF)

Figure S3 **H_2_O_2_ does not affect macrophage infiltration.** Results shown are mean ± S.E.M, n = 6–7. A representative section from each treatment is shown. ES – Eschar; HE – Hyper-proliferating epidermis; ND – neodermis.*p<0.05
(TIF)

Figure S4 **ERK and p38 phosphorylation is attenuated by 4 h.** (A) Representative blots of wound tissues lysate collected 30 min after wounding. Skin denotes skin from non-wounded animals while control refers to wounds treated with PBS. (B) The density of phosphorylated ERK and pan-ERK and (C) phosphorylated p38 and pan p38 were normalized against α-tubulin. Results shown are mean ± S.E.M. (n = 4). Densitometry results were analyzed by 1-way ANOVA and was not statistically significant.
(TIF)

Figure S5 **Wounding increases chemokine levels in wounds but 166 mM H_2_O_2_ does not further increase it.** Day 6 wound tissues were lysed and analyzed using a bead-based suspension array method. Skin denoted skin obtained from unwounded animals. (A) CXCL1 a.k.a. KC, (B) CXCL5 a.k.a. LIX, (C) CCL2, a.k.a. MCP-1 and (D) MIP-1α were strongly up-regulated after wounding but not affected by treatment with 166 mM H2O2. The results shown are the mean fold change ± S.E.M. n = 5
(TIF)

Manual S1 **ColSeg ImageJ Plug-in User Manual.**
(PDF)
