# Supplementary figures and images for: Effects of Hydrogen Peroxide on Wound Healing in Mice in Relation to Oxidative Damage
Source: PLoS One. 2012 Nov 13;7(11):e49215. doi: 10.1371/journal.pone.0049215 (PMC3496701; doi:10.1371/journal.pone.0049215)

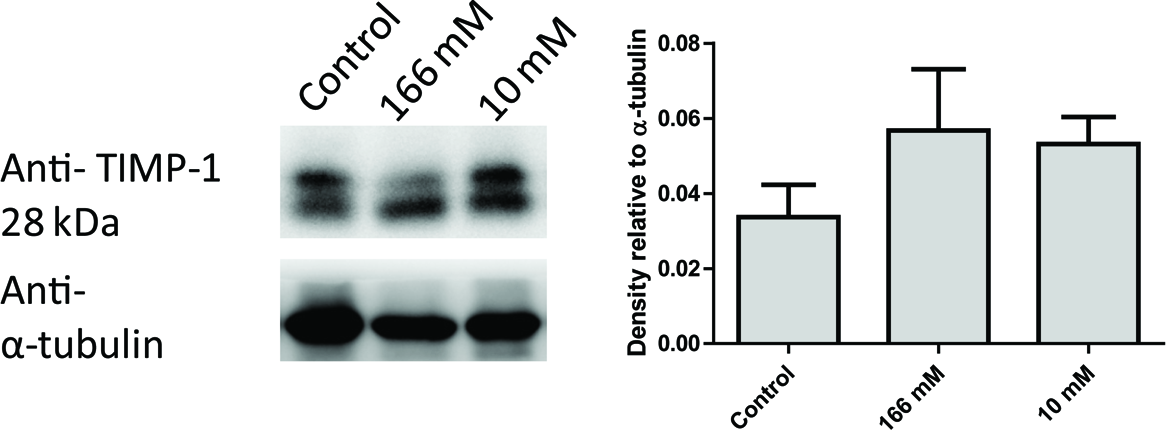

Supplement: Figure S1 — H2O2 did not affect levels of TIMP-1 in wounds. Day 6 wound tissues were lysed and the amount of TIMP-1 was measured using western blot. (A) Representative blot of TIMP-1. (B) Densitometry analysis of both bands normalized against α-tubulin. The results shown are mean ± S.E.M. (n = 4). Results were analyzed using 1-way ANOVA and the differences were not significant. (p = 0.35) (TIF) [file pone.0049215.s001.tif]

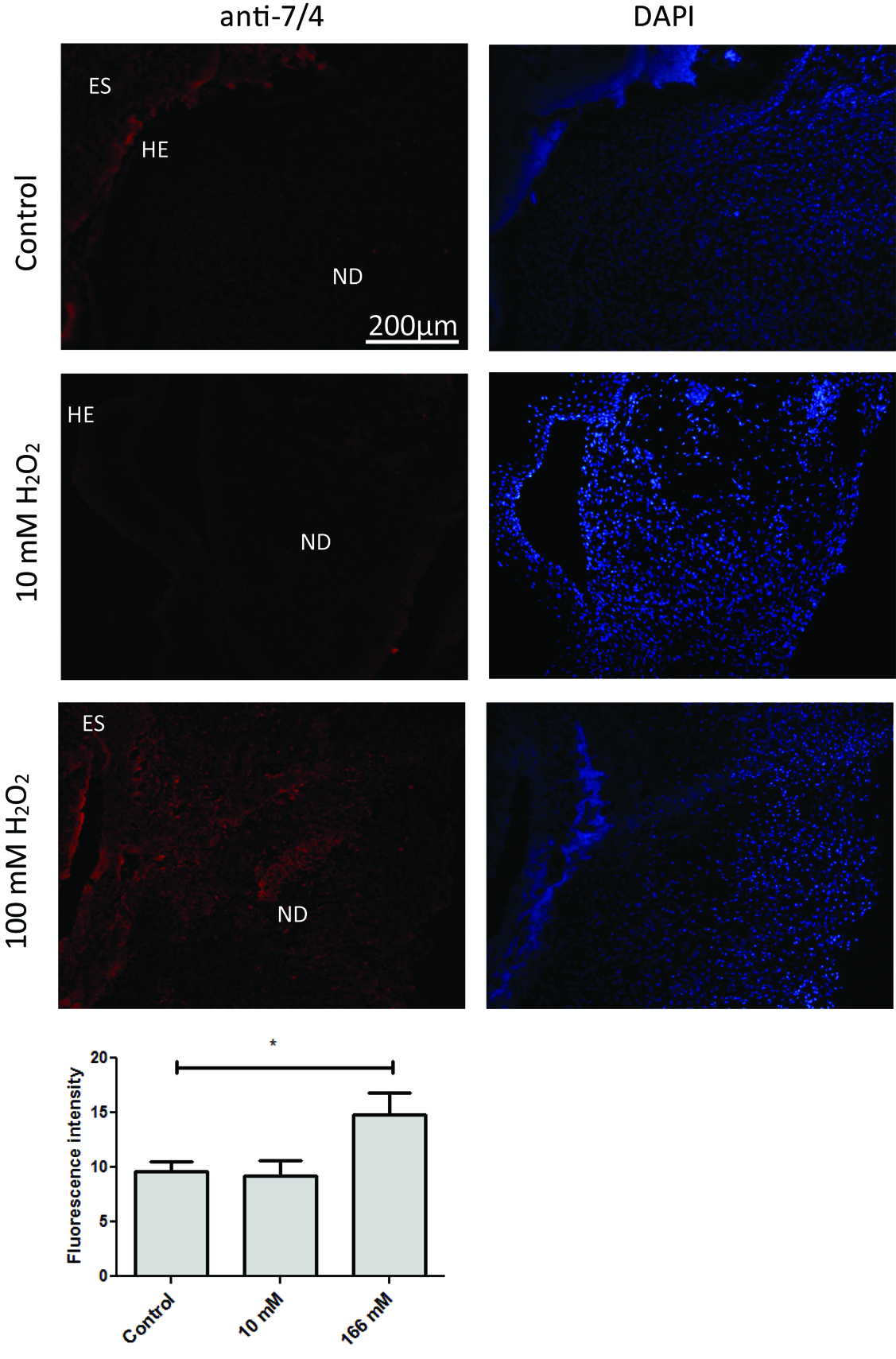

Supplement: Figure S2 — 166 mM H2O2 increased neutrophil infiltration but 10 mM H2O2 does not. Results shown are mean ± S.E.M, n = 6–7. A representative section from each treatment is shown. ES – Eschar; HE – Hyper-proliferating epidermis; ND – neodermis.*p<0.05 (TIF) [file pone.0049215.s002.tif]

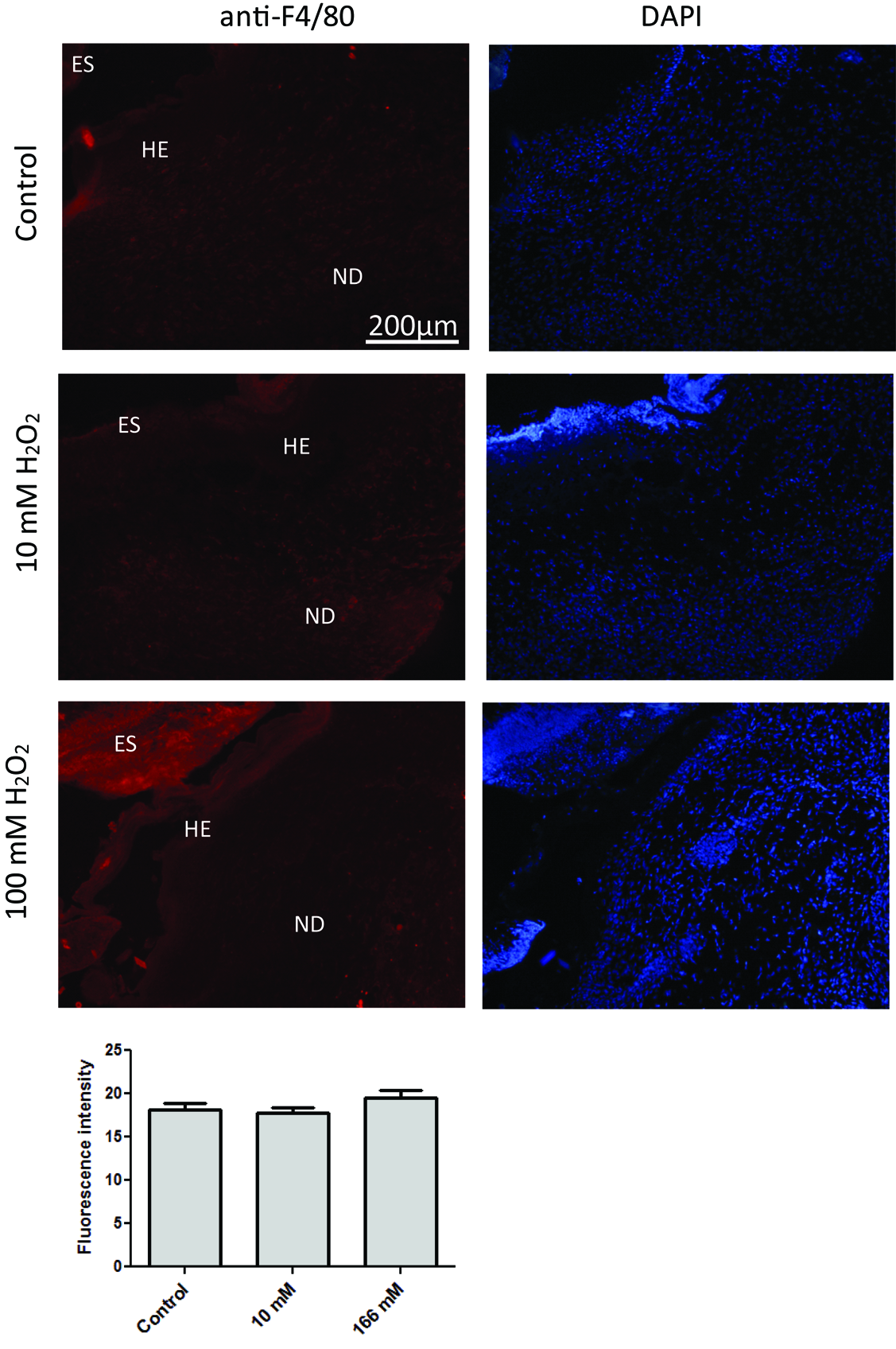

Supplement: Figure S3 — H2O2 does not affect macrophage infiltration. Results shown are mean ± S.E.M, n = 6–7. A representative section from each treatment is shown. ES – Eschar; HE – Hyper-proliferating epidermis; ND – neodermis.*p<0.05 (TIF) [file pone.0049215.s003.tif]

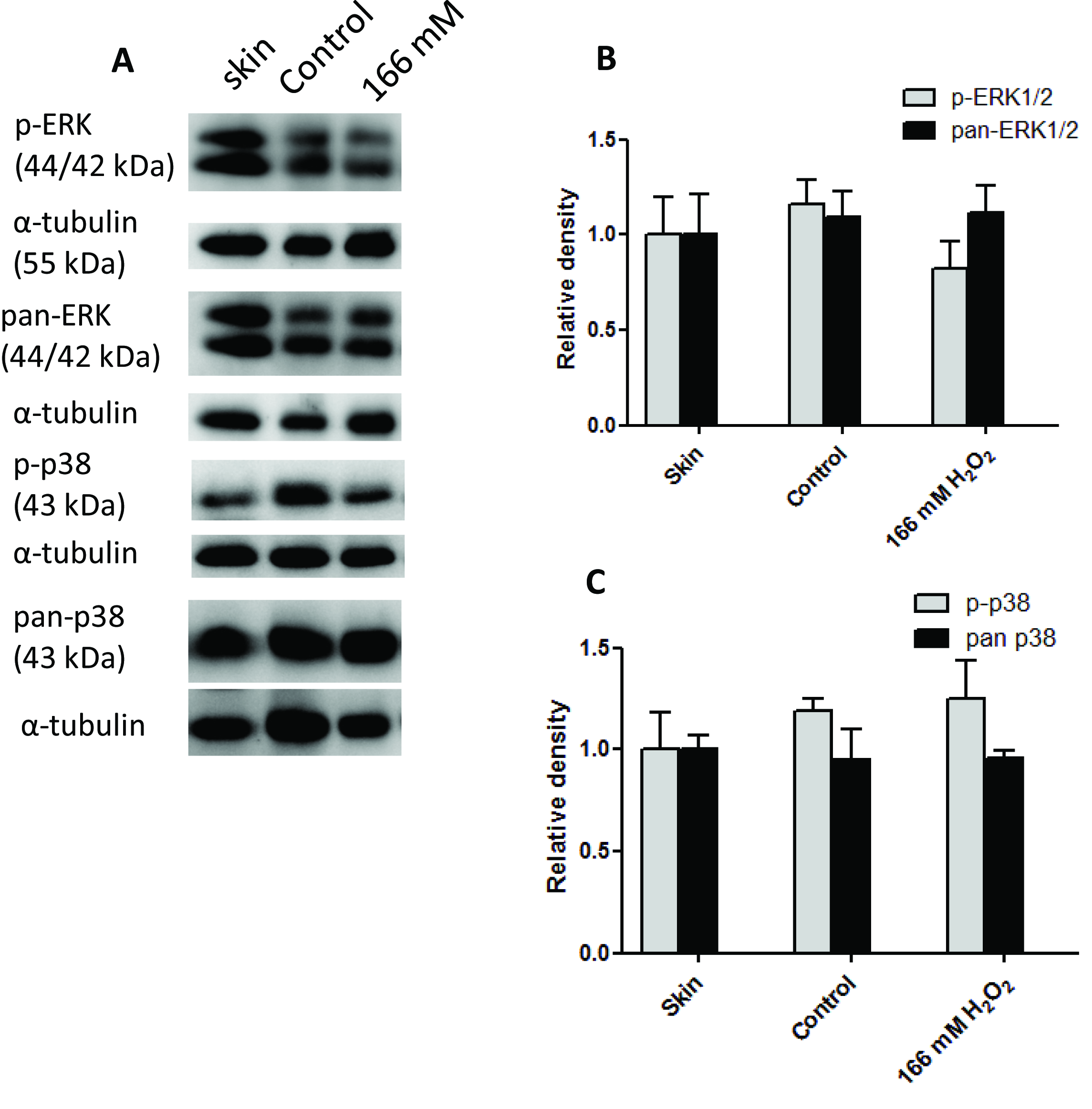

Supplement: Figure S4 — ERK and p38 phosphorylation is attenuated by 4 h. (A) Representative blots of wound tissues lysate collected 30 min after wounding. Skin denotes skin from non-wounded animals while control refers to wounds treated with PBS. (B) The density of phosphorylated ERK and pan-ERK and (C) phosphorylated p38 and pan p38 were normalized against α-tubulin. Results shown are mean ± S.E.M. (n = 4). Densitometry results were analyzed by 1-way ANOVA and was not statistically significant. (TIF) [file pone.0049215.s004.tif]

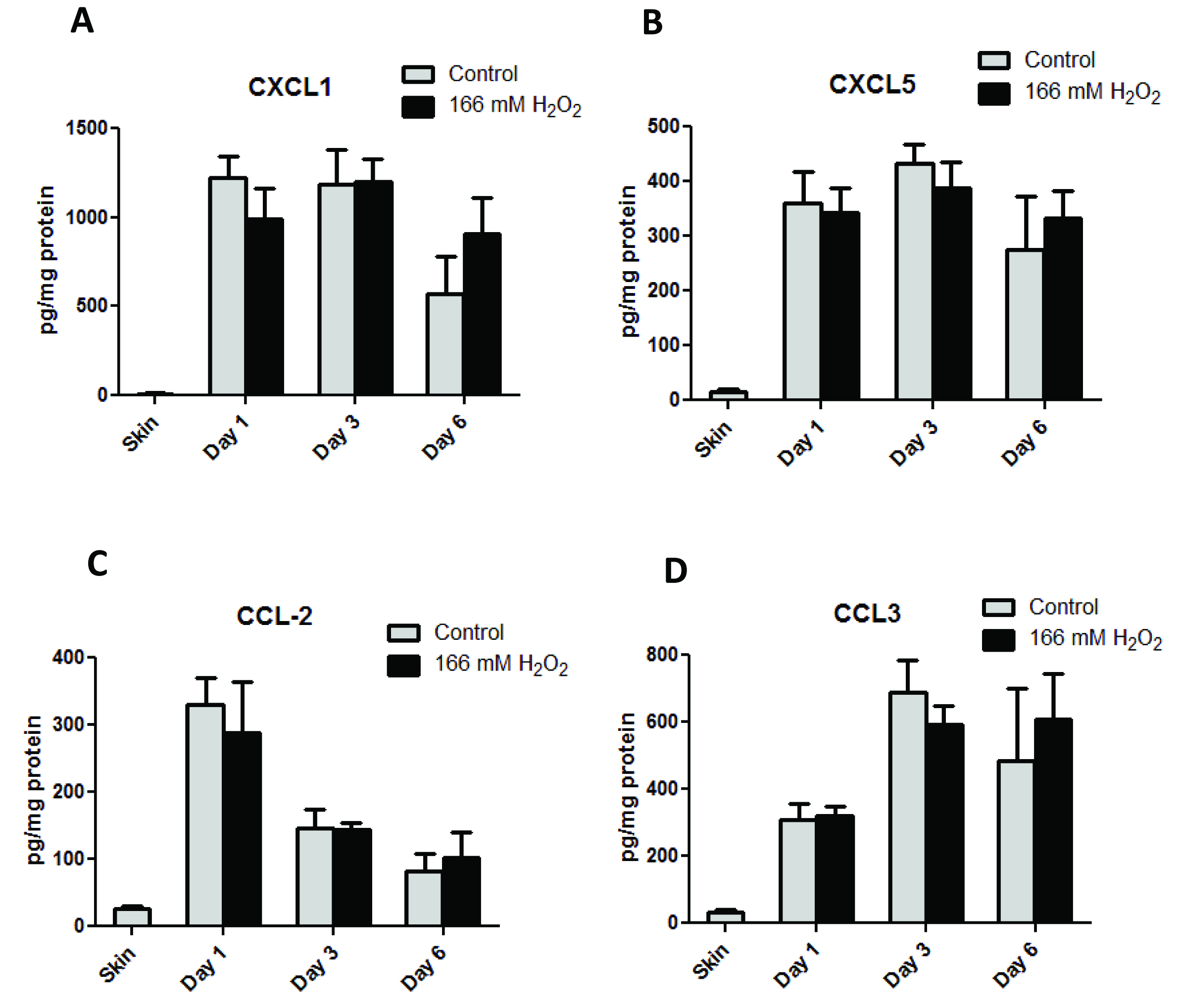

Supplement: Figure S5 — Wounding increases chemokine levels in wounds but 166 mM H2O2 does not further increase it. Day 6 wound tissues were lysed and analyzed using a bead-based suspension array method. Skin denoted skin obtained from unwounded animals. (A) CXCL1 a.k.a. KC, (B) CXCL5 a.k.a. LIX, (C) CCL2, a.k.a. MCP-1 and (D) MIP-1α were strongly up-regulated after wounding but not affected by treatment with 166 mM H2O2. The results shown are the mean fold change ± S.E.M. n = 5 (TIF) [file pone.0049215.s005.tif]
