# Supplementary material for: Effects of Hydrogen Peroxide on Wound Healing in Mice in Relation to Oxidative Damage
Source: PLoS One. 2012 Nov 13;7(11):e49215. doi: 10.1371/journal.pone.0049215 (PMC3496701; doi:10.1371/journal.pone.0049215)

# ColSeg ImageJ Plug-in User Manual

**Brief description**

This is a custom software for color image segmentation that was implemented as an ImageJ plug-in. In ImageJ, the user opens an image of the entire wound region. The software categorize all pixels into one of three colors, namely green, red and white. The user chooses up to 5 representative pixels within an image to train the software to recognize the color selected. A region of interest (ROI) can be selected using the "Polygon Selection" to spatially restrict image processing. Without ROI selection, pixel categorization is applied to the whole image.

During processing, the image is transformed from the RGB into the CIELAB color space, which was designed to provide a better approximation of human color perception than RGB. Each pixel in the ROI is assigned a class label (green, red or background) based on similarity to the mean color of the sampled pixels in the CIELAB color space. Lastly, the software identify connected pixels with the same class as a segment.

The "Result" panel displays the statistical analysis of the image segmentation, including total number of pixels of each of the three colors, and number and average size of the segments. To ensure reproducibility, the parameters for color selection was saved and reused for other images.

**Prerequisites**

• Download and install ImageJ or FIJI on your computer system

• Make sure that the "Gray_Morphology.jar" plugin file is present in the plugins folder. If not, download from http://rsbweb.nih.gov/ij/plugins/gray-morphology.html

• Download the "colseg_.jar" file from the following URL: http://web.bii.a-star.edu.sg/archive/colseg/ and install in the ImageJ plugins folder

**Color Image Segmentation**

- Open ImageJ
- Open (stitched) color image.

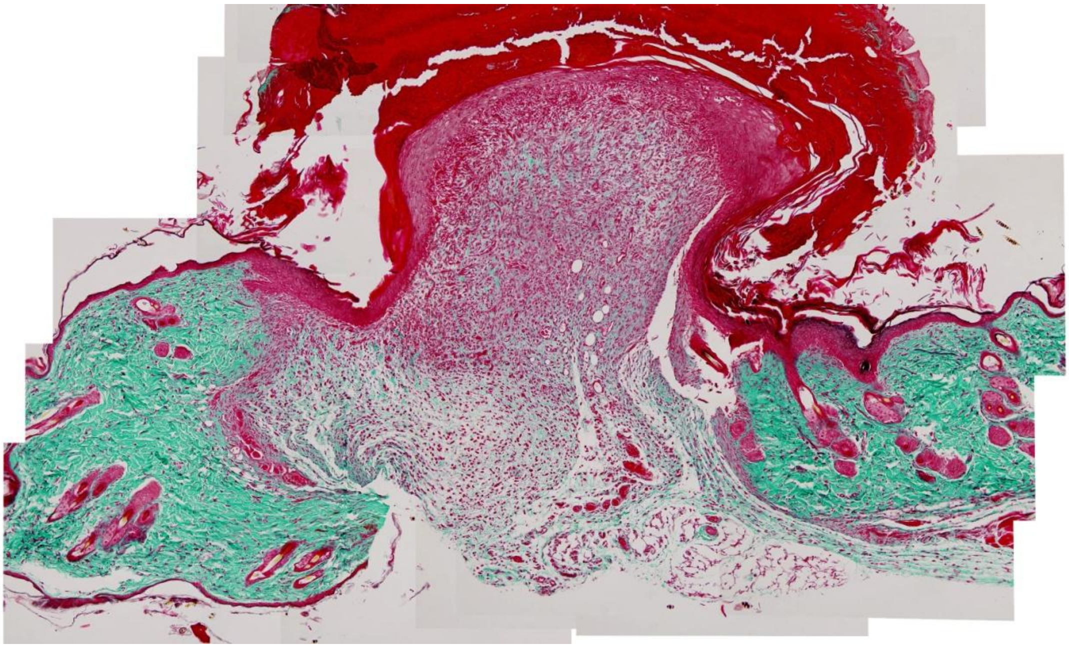

• Open ColSeg using Menu -> Plugins -> ColSeg

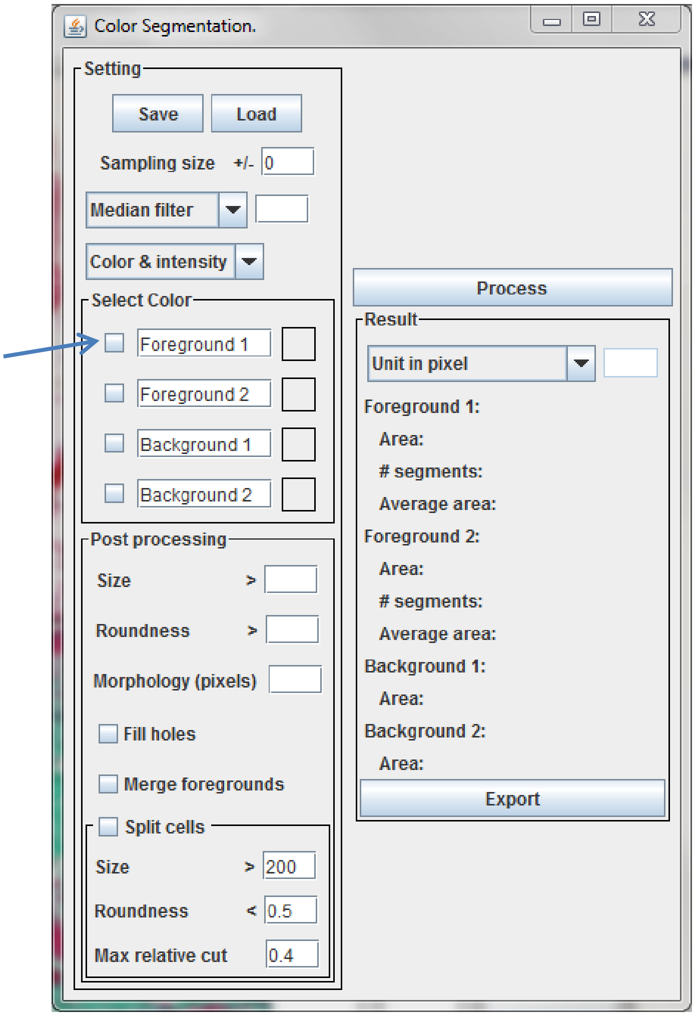


• In the Select Color box, choose one of the color classes for which define representative pixels by clicking on CheckBox (blue arrow). Here we assume, that the user needs to distinguish between three classes of pixels; two foreground (red and green) and one background class. It is possible to change the names of the labels.
• Inside the color image, select up to 5 representative points, which will be shown in a separate dialog (below). Repeat this step for all classes of pixels.
• Color selection profiles can be saved and loaded for the segmentation of other images.

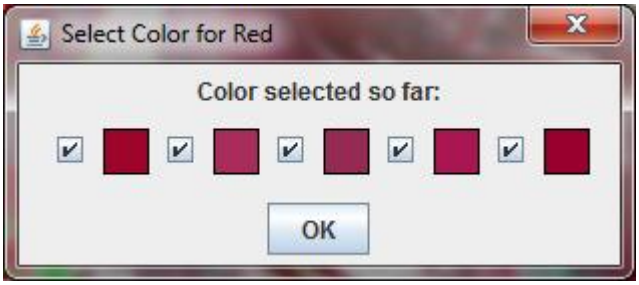

• (optional) use the “Polygon Selection” tool to select a region of interest (ROI)

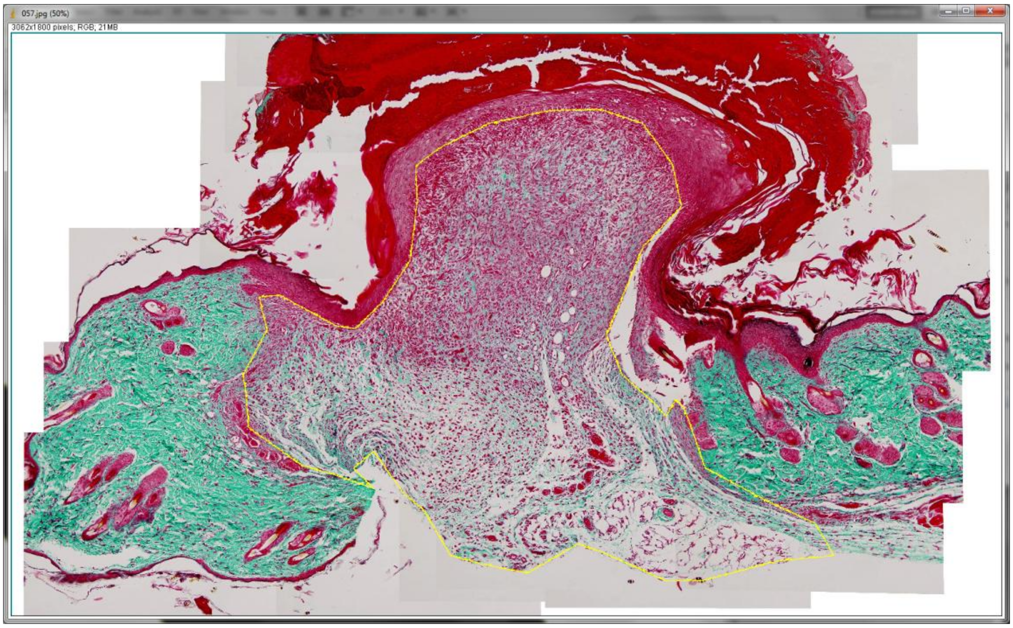


• Press the “Process” button to segment the image. The pixels inside the image (ROI) are assigned to any of the three classes according to similarity to their representative pixels in the LAB color space.

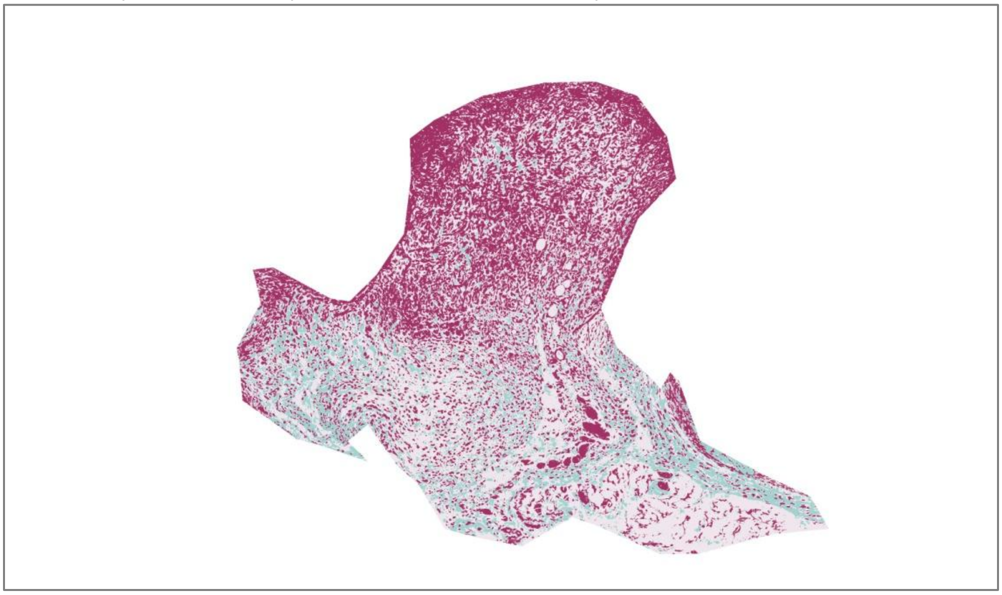

• The total area for each class the number of segments is shown in the Result box. The statistics can be exported as a text file.

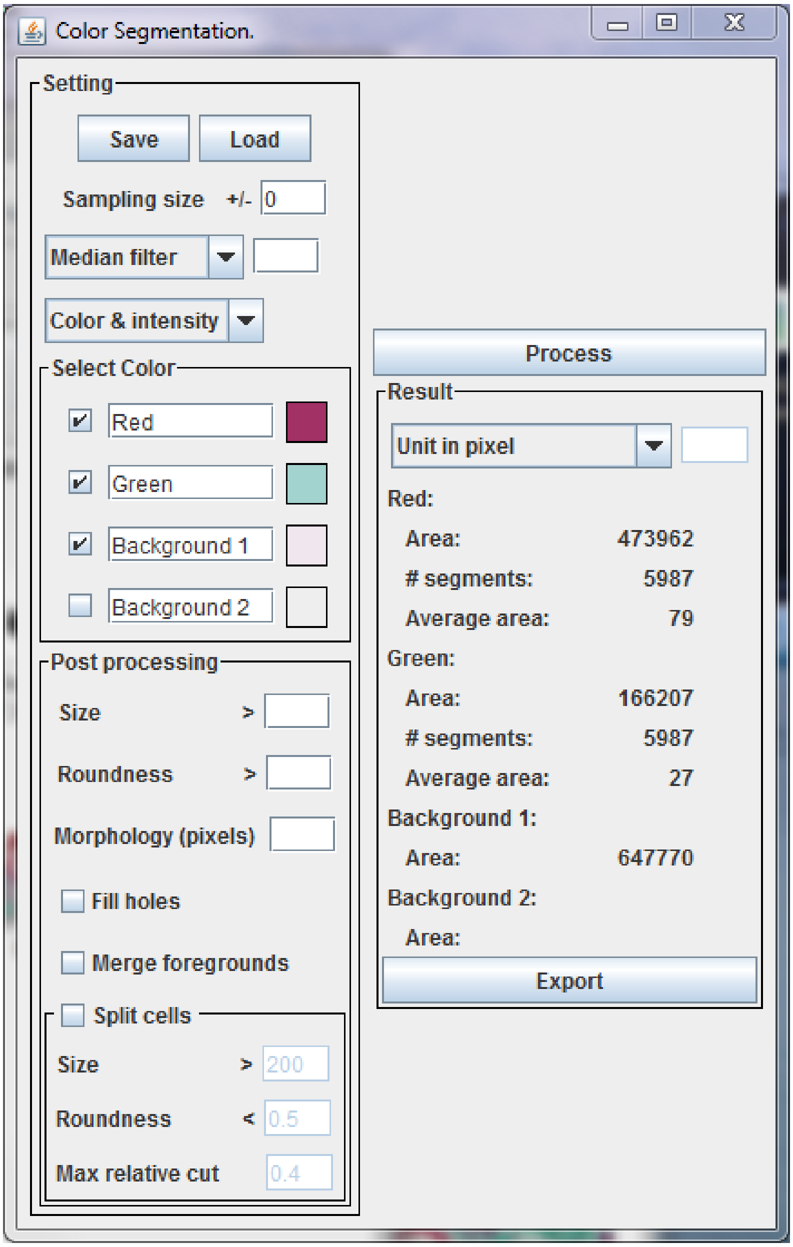

Supplement: Manual S1 — ColSeg ImageJ Plug-in User Manual. (PDF) [file pone.0049215.s006.pdf]
